# Sexual Compatibility among Different Host-Originated Isolates of *Aphelenchoides besseyi* and the Inheritance of the Parasitism

**DOI:** 10.1371/journal.pone.0040886

**Published:** 2012-07-18

**Authors:** Shu-Hua Hsieh, Chun-Ju Lin, Peichen Chen

**Affiliations:** Department of Plant Pathology, National Chung Hsing University, Taichung, Taiwan; Nanjing Agricultural University, China

## Abstract

Nine isolates of *Aphelenchoides besseyi* from two different hosts were studied. The isolates were identified at the species level according to morphometrics and fine structures observed under a scanning electron microscope. Two fern-originated isolates, Fu, and Fm, one rice-originated isolate, Rl, were not able to reproduce from a single juvenile, based on at least 50 replicates. The other six isolates were able to develop into a small population when inoculated with a single juvenile, demonstrating parthenogenesis. Crosses between isolates were conducted. In a compatibility cross experiment, three fern-originated isolates were selfed and crossed reciprocally, and all nine crossings had viable offspring. When fern isolates were used as paternal lines, the only two successful crosses were with the Rd line, and as maternal lines, only the Ff x Re and Fu x Rn crosses had viable offspring. Rl was used as the maternal line and Fm as the paternal line to study the inheritance of the bird’s-nest fern parasitism. Twenty of the 80 attempted crosses resulted in viable offspring and among these; six lines had the ability to parasitize on the bird’s-nest fern. When the F_1_ lines were back-crossed to the Rl maternal line, 20 viable offspring lines were obtained and among them 4 were able to parasitize bird’s-nest fern. These results indicate that bird’s-nest fern parasitism can be transferred to new generations by cross fertilization.

## Introduction

Within the plant-parasitic *Aphelenchoides* genus, four species are well recognized: *A. besseyi* Christie, 1942; *A. fragariae* Christie 1932; *A. ritzemabosi* Steiner and Buhrer, 1932; and *A. arachidis* Bos, 1977. The most common and economically important *Aphelenchoides* species in Taiwan is *A. besseyi* [1], [2], [3]. *A. besseyi* has been reported to parasitize rice (*Oryza sativa* L.), strawberry (*Fragaria grandiflora* Ehrn.), bird’s-nest fern (*Asplenium nidus* L.), and many other ornamental plants [2], [3], [4]. Rice fields with infection rates of 34–58% resulted in a 35–45% reduction in yield due *A. besseyi* [1]. The bird’s-nest fern is an economically important perennial vegetable crop in Taiwan and *A. besseyi* was commonly found on farms that grow this fern [3].

In the *Aphelenchoides* genus, *A. ritzemabosi*, *A. fragariae*, *A. blastophthorus* and *A. composticola* have been documented to reproduce by cross fertilization [5], [6], [7], [8], [9]. *A. bicaudatus* reproduction occurs by meiotic parthenogenesis [10]; however, the mode of reproduction in *A. besseyi* is controversial. Sudakova and Stoyokov (1967) reported that reproduction occurred by parthenogenesis [11], while Franklin and Siddiqi (1972) [12] documented *A. besseyi* reproduction as amphimictic. Huang et al. (1979) showed in single juvenile inoculation tests that the *A. besseyi* tested were amphimictic [13]. In Taiwan, *A. besseyi* was also originally reported as an amphimictic species [2], [14]. However, Tzen (2003) recently reported the occurrence of both parthenogenesis and amphimixis among four isolates of *A. besseyi* [15].

Variation in virulence and aggressiveness has been observed in many strains of both *A. fragariae* and *A. ritzemabosi* from different hosts and localities. This variation indicates great intraspecific heterogeneity, and might lead to variation in host range [16]. Noegel and Perry found that *Aphelenchoides besseyi* isolated from *Chrysanthemum* could not infect strawberry [17]; Rigges (1991) reported that *A. besseyi* isolated from strawberry could not infect rice, and vice versa [18]. These results show that *A. besseyi*, like *Ditylenchus dipsaci*, has different host preferences between isolates [19], but the existence of races of *A. besseyi* was not mentioned as a problem because the races were not differed by economically important hosts [17]. In Taiwan, *A. besseyi* was first reported on bird’s-nest fern (*Asplenium nidus* L.) in 2002 and caused dark-brown patches on the leaves. This isolate was also capable of infecting strawberry and rice [3]. In the same study, a strawberry isolate was found to parasitize both rice and strawberry, but parasitism of the rice isolates was restricted to rice. Regardless of their ability to infect different hosts, these isolates did not differ in their morphology [3].

**Table 1:** The number of propagating replicates from single juvenile cultures of nine *Aphelenchoides besseyi* isolates on *Alternaria citri*.

Fern Line	Propagating ratio	Rice Line	Propagating ratio
Ff	20/20	Rl	0/100
Fu	0/100	Rp	4/20
Fk	5/100	Re	20/20
Fm	0/50	Rn	8/20
		Rd	4/20

There have been no studies about the relationship between the *Aphelenchoides besseyi* host races and the mode of reproduction, or any attempts to cross isolates of *A. besseyi* to study the inheritance of the parasitism traits. The objectives of this study were (i) to conduct a cross fertilization experiment with *A. besseyi* isolates collected from different hosts and observe their compatibility, and (ii) to study the inheritance pattern of parasitism in *A. besseyi* by crossing rice and fern isolates.

## Results

### Identification

The average morphometric measurements from the Fm isolate and the eight isolates obtained during 2004–2006, were within the range of measurements of the *Aphelenchoides besseyi* topotype described by Franklin and Siddiqi 1972 (data not shown). All nematodes had an off-set lip region, three to four pointed processes on the tail and four lateral incisures when observed under the cryo-field emission scanning electron microscope (data not shown). These characteristics were sufficient to support the species level identification [20].

**Table 2:** The number of surviving and dying offspring lines from crosses of *Aphelenchoides besseyi* isolates.

Cross	L^a^	D^a^	Cross	L^a^	D^a^	Cross	L^a^	D^a^
Ff♀×Ff♂	7	1	Ff♀×Fu♂	4	2	Ff♀×Fk♂	5	1
Fu♀×Ff♂	7	1	Fu♀×Fu♂	3	3	Fu♀×Fk♂	6	0
Fk♀×Ff♂	3	3	Fk♀×Fu♂	5	1	Fk♀×Fk♂	5	1
Rl♀×Ff♂	0	6	Rl♀×Fu♂	0	6	Rl♀×Fk♂	0	6
Rp♀×Ff♂	0	6	Rp♀×Fu♂	0	6	Rp♀×Fk♂	0	6
Re♀×Ff♂	0	6	Re♀×Fu♂	0	6	Re♀×Fk♂	0	6
Rn♀×Ff♂	0	6	Rn♀×Fu♂	0	6	Rn♀×Fk♂	0	6
Rd♀×Ff♂	0	6	Rd♀×Fu♂	5	1	Rd♀×Fk♂	2	4
Ff♀×Rl♂	0	6	Fu♀×Rl♂	0	6	Fk♀×Rl♂	0	6
Ff♀×Rp♂	0	6	Fu♀×Rp♂	0	6	Fk♀×Rp♂	0	6*
Ff♀×Re♂	1	5*^b^	Fu♀×Re♂	0	6	Fk♀×Re♂	0	6
Ff♀×Rn♂	0	6	Fu♀×Rn♂	1	5*	Fk♀×Rn♂	0	6
Ff♀×Rd♂	0	6	Fu♀×Rd♂	0	6	Fk♀×Rd♂	0	6

a L: the number of crosses that produced offspring, D:the number of crosses that did not propagate.

b *: two to four crosses produced offspring that failed to survive.

### Modes of Reproduction

Two fern-originated isolates, Fu and Fm, and one rice-originated isolate, Rl, did not reproduce from a single juvenile in at least 50 replicates observed in this study. The other six isolates had replicates that were able to develop into a small population, demonstrating the ability to reproduce by parthenogenesis (Table 1). Among these parthenogenetic isolates, the Fk, Rp, Rn and Rd isolates had a lower percentage of replicates that were able to develop into a population from a single juvenile (Table 1).

### Compatibility between *Aphelenchoides besseyi* Isolates

The fern-originated isolates were selfed and crossed reciprocally, and all nine combinations had viable offspring (Table 2). When fern-originated isolates were used as the paternal line and five rice-originated isolates as the maternal line, only the Rd x Fu and Rd x Fk crosses resulted in viable offspring. When fern-originated isolates were used as the maternal line and five rice-originated isolates were used as paternal lines, only the Ff x Re and Fu x Rn crosses had viable offspring (Table 3). When fern isolates were used as paternal lines, the only two successful crosses were with the Rd line yielding 33–83% viable offspring. The lowest rate of viable offspring was 17% when fern isolates were used as maternal lines (Table 2).

**Table 3:** Successful crosses of Rl(♀) and Fm(♂) and their bird’s-nest fern pathogenicity.

Batch number	The number of non-infectious F_1_ lines	The number of infectious F_1_ lines (code)
1	6	**2** (1-1,1-2)
2	8	**0**
3	0	**1** (3-1)
4	0	**3** (4-1,4-2,4-3)
5	– a	NA b
6	–	NA
7	–	NA
8	–	NA
**Total number**	**14**	**6**

a No viable F_1_ resulted from the crosses in this batch, and each batch contained 10 crosses.

b Not applicable.

**Figure 1:**
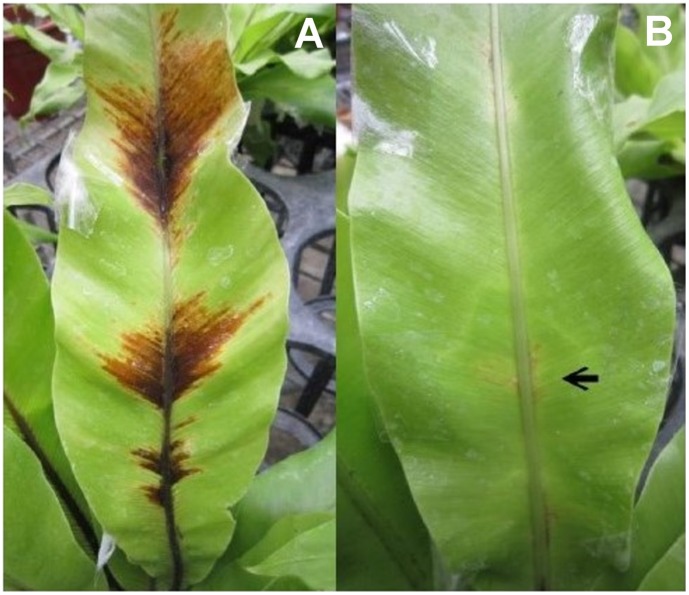
The bird’s-nest fern leaves inoculated with different F_1_ lines. (A) The bird’s-nest fern leaf was inoculated with the infectious F_1_ line (1-1) from the Rl x Fm cross. (B) No symptoms were observed on bird’s-nest fern leaf when inoculated with none-infectious F_1_ line (2–8) from the Rl x Fm cross. The arrow indicated the injection site.

### Inheritance of Bird’s-nest Fern Parasitism in *Aphelenchoides besseyi*

The first four batches of crosses between the Rl and Fm lines had 20 viable F_1_ lines (Table 3), but batches four to eight did not result in any F_1_ lines. When the 20 F_1_ lines were used to inoculate a bird’s-nest fern, lines 1-1, 1-2, 3-1, 4-1, 4-2 and 4-3 had the ability to parasitize fern (Table 3), causing typical symptoms to appear on the leaves (Fig 1A), in contrast with the F_1_ lines that could not parasitize the fern, and did not show any symptoms (Fig 1B).

When the six F_1_ lines were back-crossed to the Rl maternal line, 120 attempted crosses resulted in 20 viable back-cross lines (Table 4). The back-cross of F_1_ 1-1×Rl had 8 viable offspring lines, and F_1_ 1-2×Rl had 5 lines. The F_1_ 4-1, F_1_ 4-2 and F_1_ 4-3 back-crosses resulted in only one offspring line. The back-cross of F_1_ 3-1×Rl did not result in any viable offspring. When the back-cross lines were inoculated on the bird’s-nest fern, lines 11.7, 12.2, 31.1 and 42.1 were able to parasitize the fern (Table 4).

**Table 4:** Backcrosses of bird’s-nest fern infectious F_1_ lines(♂) to a maternal Rl line(♀) originated from rice.

Paternal F_1_ line code	The number of non infectious back cross lines	The number of bird’s-nest fern infectious back cross lines (code)
1-1	8	**1** (11.7)
1-2	5	**1** (12.2)
3-1	0	**1** (31.1)
4-1	1	**0**
4-2	1	**1** (42.1)
4-3	1	**0**
**Total number**	**16**	**4**

## Discussion

All nine isolates in this study were identified as *A. besseyi* based on morphometric data. However, the isolates varied in their abilities to infect the bird’s-nest fern. The fern-originated isolates could parasitize strawberry and rice, but the rice-originated isolates could not parasitize fern [3], [15]. Because nematode populations within plants are relatively isolated and often small, the chances are fairly high that favored genetic factors will increase and lead to intraspecific diversity. Almost all known cases of intraspecific variation in pathogenicity are confined to endoparasitic species [16]. *A. besseyi* is ectoparasitic for most of its life cycle in the majority of its hosts [7], [12], and distinct food preferences between the isolates is a unique phenomenon.

In this study, most but not all isolates were able to reproduce from a single juvenile, indicating that some isolates might reproduce by cross fertilization, while the others have already evolved parthenogenesis. These results not only confirmed that the *A. besseyi* species has more than one mode of reproduction, but also showed two reproductive modes in one host race for the first time. Nematodes that belong to the same species but have two modes of reproduction are rare. *Meloidogyne hapla* has two biological races. Race A reproduces by facultative parthenogenesis and race B by obligate parthenogenesis [21], [22]. The differences between these two races were found in the cytology of their ovaries. Facultative parthenogenesis involves meiotic division and obligate parthenogenesis uses mitotic division. In the studies of *Meloidogyne* genus, it was believed that species with sexual reproduction were more primitive than asexual ones [22]. Cayrol and Dalmasso (1975) studied the interspecific relationships among *A. besseyi*, *A. ritzemabosi* and *A. fragariae* and reported that crossing one juvenile from one of the species and ten males from another species gave positive results for five of the six possible crosses, and intermediate characteristics were observed in offspring [17]. Whether the species of the *Aphelenchoides* genus are of mixed origins like the *Meloidogyne* genus [23] and are in a transitional stage with both obligate cross fertilization and facultative (or obligate) parthenogenesis remains unanswered until further cytogenetic analysis of the nematodes can be performed.

In cross compatibility experiments, several combinations of crosses between fern- and rice- originated lines did not result in viable offspring; only four combinations produced a total of nine viable offspring lines (Table 2). On the other hand, 38 crosses were made from six combinations of the fern-originated isolates and resulted in 30 viable offspring lines, suggesting that the reproductive barrier between the nematode isolates from different hosts might form gradually. Organisms that move slowly may have developed into a new species after migrating to a new niche (parapatric speciation). Some parasites exhibit interspecific polymorphism, allowing individuals with a certain genotype to adapt to a new host (sympatric speciation) [24]. We observed that the Ff and Re isolates were able to reproduce vigorously by parthenogenesis (Table 1) but did not produce any viable offspring when their progenies were crossed (Table 2). Similar results were observed in the Fk, Rn and Rp lines, indicating that parthenogenesis was inhibited by the presence of a male individual. The mechanism for this interesting phenomenon deserves further investigation.

The study of the inheritance of bird’s-nest fern parasitism of *A. besseyi* is the first to be attempted in this genus. The parasitism behavior of above ground nematodes is quite different from that of underground, endoparasitic nematodes, such as *Meloidogyne* and *Globodera* spp. [25]. Unlike these obligate parasitic nematodes, the *Aphelenchoides* spp. are able to feed on fungal hyphae when host plants are not available, suggesting that the facultative parasitic *Aphelenchoides* spp. might use a very different strategy to cause plant diseases. Although a large number of crosses were made in this study, only a few viable F_1_ offspring lines were obtained. Among them, we observed the cross between bird’s-nest fern non-infectious Rl and bird’s-nest fern infectious Fm resulted in viable offspring that were also able to parasitize bird’s-nest fern. When these bird’s-nest fern infectious F_1_ lines were backcrossed to the Rl maternal line, four viable offspring lines inherited the ability to infect bird’s-nest fern. These results indicate that bird’s-nest fern parasitism can be transferred to new generations by cross fertilization.

## Materials and Methods

### Collection of *Aphelenchoides besseyi* Isolates

Eight isolates of *Aphelenchoides besseyi* from two different hosts were studied during 2004–2006 (Table 5). The Fm isolate was obtained 2009, and therefore not included in the compatibility test. *A. besseyi* isolates were collected from the infected rice grain or fern leaves. Diseased tissue was washed several times with distilled water to clean the surface, immersed in distilled water, and cut coarsely to release the nematodes. Nematodes were observed after 5–10 min and transferred onto PDA slants containing *Alternaria citri*. The nematode cultures were maintained at 24°C without light to increase the population. The isolates were identified at the species level. Nematodes were sterilized with 1000 ppm malachite green and 2000 ppm streptomycin, and maintained on *Alternaria citri* PDA slants [3] in a 24°C growth chamber.

**Table 5:** The codes, and original hosts of the nine *Aphelenchoides besseyi* isolates in this study.

Line	Original host	Locality
Ff	*Asplenium nidus* L., Bird’s-nest fern	Chiayi county
Fu	*Asplenium nidus* L., Bird’s-nest fern	Sansia, Taipei county
Fk	*Asplenium nidus* L., Bird’s-nest fern	Shengang, Taichung county
Fm	*Asplenium nidus* L., Bird’s-nest fern	Mingjian, Nantou county
Rl	*Oryza sativa* L., paddy rice	Linnei, Yunlin county
Rp	*Oryza sativa* L., paddy rice	Beidou, Changhua county
Re	*Oryza sativa* L., paddy rice	Erlin, Changhua county
Rn	*Oryza sativa* L., paddy rice	Chiunglin, Hsingchu county
Rd	*Oryza sativa* L., paddy rice	Dajia, Taichung county

### Identification of Nematode Isolates

From each uncharacterized isolate, available male and 30 female nematodes were measured under the light microscope according to De Man’s formula. Typical characterization features were photographed. Fine structures such as lateral incisures and the number of tail processes were observed under a cryo-field emission scanning electron microscope (JEOL6330 Cryo-FESEM, JEOL Ltd., Tokyo, Japan). A portion of a 20 µl nematode suspension containing a few hundred individuals was dispensed onto 2 sheets of Kimwipes® EX-L (Kimberly-Clark Corporation, Georgia, U.S.A.), and the excess water removed. The Kimwipes were glued onto the metal specimen stage, and protected with a metal cover. The samples were fixed in liquid nitrogen and sent into the vacuum specimen chamber within 90 seconds for observation. The images were saved as JPG digital files with a resolution of 1280×1024 pixels.

### Analysis of Reproduction Modes of *Aphelenchoides besseyi* Isolates

Before the crossing experiments could be performed, the mode of reproduction of the *A. besseyi* isolates needed to be clarified. Single juveniles were transferred onto *Alternaria citri* PDA slants, with at least 20 replicates for each isolate. The cultures were incubated without lights at 24°C for 14 to 28 days before examination. If a single juvenile was able to reproduce, multiple nematodes would be present in the PDA slant tube, indicating that an isolate was capable of reproducing parthenogenetically. More replicates were performed if the isolates did not reproduce in the first 20 replicates. A maximum of 100 replicates were performed.

### Compatibility between *Aphelenchoides besseyi* Isolates Originated from Different Hosts

Three bird’s-nest fern isolates were selfed and crossed reciprocally for nine combination crosses. The three bird’s-nest fern isolates, Ff, Fu and Fk, and five isolates from rice were used as parental lines. A total of 30 combination crosses were conducted, and all crosses had six to eight replicates. One juvenile from the maternal line and one adult male from the paternal line were transferred onto a *Alternaria citri* PDA slant to conduct the cross fertilization. The crosses were maintained under the same conditions described in the previous paragraph and observed after 14–28 days. All of the cultures were observed under a dissecting microscope. If no nematodes were observed, some distilled water was added to the tube and vortexed several times to wash the culturing slant for further observation.

### Inheritance of the Bird’s-nest Fern Parasitism in *Aphelenchoides besseyi*

Based on the results from the reproduction mode experiments and the previous host range studies, the Rl isolate from rice and the Fm isolates from bird’s-nest fern were chosen for the cross. Rl which could not infect bird’s-nest fern was used as the maternal line, and Fm, which could infect bird’s-nest fern, was used as the paternal line to ensure that the offspring that obtained the parasitism trait was from a successful cross. Eight batches of crosses were conducted at different times and 10 crosses were made in each batch. The viable offspring were maintained on *Alternaria citri* PDA slants to increase nematode population, and challenged on bird’s-nest fern by injecting approximately 1000 nematodes into the leaf [3]. The F_1_ lines that were able to infect bird’s-nest fern were used as paternal lines and Rl was used as the maternal line in the back-crosses. For each combination, 20 crosses were made. All viable backcross lines were maintained on *Alternaria citri* PDA slants to increase nematode population and challenged on the bird’s-nest fern to determine their parasitic ability.
